# Exploration of heterogeneity in risk factors associated with imaging subtypes of white matter hyperintensities on fluid-attenuated inversion recovery magnetic resonance imaging

**DOI:** 10.3389/fneur.2025.1647065

**Published:** 2025-08-26

**Authors:** Yonglong Li, Xiufu Zhang, Haotian Wang, Linrong Jiang, Zhaoyang Gu, Jun Zhou, Ruipeng Liang

**Affiliations:** Department of Radiology, Jiangjin Central Hospital of Chongqing, Chongqing, China

**Keywords:** white matter hyperintensity, risk factors, deep white matter hyperintensity, periventricular white matter hyperintensity, fluid attenuated inversion recovery

## Abstract

**Background:**

White matter hyperintensity (WMH), a critical early biomarker in cerebrovascular/neurodegenerative diseases, has traditionally been studied via global volume or subjective scoring, which overlooks its spatial heterogeneity, leading to conflicting risk factor conclusions. Recent neuroimaging advances enable “subtype resolution” research, but standardized assessments remain lacking. This study evaluates WMH risk factor spatial variability and constructs a risk stratification model to support precision prevention.

**Methods:**

This study retrospectively enrolled inpatients and outpatients aged ≥40 years [median 70.0, (59.0–77.0)] who underwent head MRI examinations due to neurological symptoms or suspected cerebrovascular disease between January 2023 and December 2024.excluding those with imaging contraindications, intracranial masses, or technical artifacts. Data included demographics (age, sex), medical history (hypertension, diabetes), and lab markers (creatinine, cystatin C). FLAIR MRI (3.0 T United Imaging uMR780) was used to acquire images. WMH volume and Fazekas scores were automatically quantified via the United Imaging AI module (UAI. OCR, R001) and validated by two senior neuroradiologists. Stratification included semi-quantitative Fazekas scoring (PWMH:periventricular WMH, DWMH:deep WMH) and anatomical segmentation (4 subregions: ventricular, periventricular, DWMH, juxtacortical). Statistical methods included Mann–Whitney U and chi-square tests for group comparisons, binary logistic regression for risk factors of moderate–severe WMH (Fazekas2-3), and multiple linear regression for volume associations (*p* < 0.05 significant).

**Results:**

Compared with absent or mild WMH (Fazekas 0–1), Group comparisons revealed that advanced age, hypertension, and abnormal renal function markers [creatinine, cystatin C, β2-microglobulin (β2-MG)] were common risk factors for moderate–severe WMH (all *p* < 0.0001). The prevalence of coronary heart disease was higher in the moderate–severe PWMH group than in the absent or mild group (22.9% vs. 12.3%, *p* = 0.001). In contrast, the moderate-to-severe DWMH group exhibited higher rates of smoking (40.3% vs. 30.2%), alcohol consumption (35.6% vs. 26.1%), and diabetes (47.0% vs. 34.8%) compared with the absent or mild group, while the prevalence of hyperlipidemia was lower (42.95% vs. 52.43%, *p* = 0.04). Multivariate models revealed that moderate–severe PWMH driven by age (OR = 1.09/year), hypertension (OR = 2.92), creatinine (OR = 2.07); moderate–severe DWMH by age (OR = 1.034/year), hypertension (OR = 2.10), smoking (OR = 1.98), diabetes (OR = 1.55), β2-MG (OR = 1.79). Cys-C (OR = 0.52) and hyperlipidemia (OR = 0.66) inversely associated with moderate–severe PWMH and moderate–severe DWMH, respectively (*p* < 0.05). Linear regression analysis demonstrated that age and hypertension strongly affected PWMH volume (*β* = 0.236–3.618); diabetes expanded periventricular lesions (*β* = 3.073); coronary heart disease and creatinine increased juxtacortical WMH (*β* = 0.232–0.280); and hyperlipidemia was inversely correlated with DWMH (*β* = −0.783) and juxtacortical WMH (*β* = −0.194) (all *p* < 0.05).

**Conclusion:**

WMH exhibits spatial heterogeneity with distinct mechanisms: PWMH associates with coronary/renal issues; DWMH with smoking/diabetes. Spatial classification optimizes risk stratification, guiding subtype-specific interventions and individualized prevention for cerebral small vessel disease.

## Introduction

White matter hyperintensity (WMH), also known as leukoaraiosis or white matter lesions, exhibits characteristic hyperintensity on T2-weighted magnetic resonance imaging (MRI) (1). Emerging evidence from evidence-based medicine suggests that WMH may serve as a critical node in the early biomarker network of cerebrovascular and neurodegenerative diseases (2–4). Thus, accurately identifying the pathogenic risk factors of WMH and establishing early intervention pathways hold significant clinical value.

Previous studies have largely treated WMH as a single pathological entity, with age and hypertension identified as core drivers based on whole-brain WMH volume or semi-quantitative scoring systems (e.g., Fazekas scale) (5–7). However, research on WMH risk factors has yielded heterogeneous conclusions (8–10), particularly regarding the relationships between WMH and diabetes (11, 12), as well as laboratory indicators (13, 14). These inconsistencies, potentially attributed to differences in study populations, sample sizes, observed indicators, and grouping methods, highlight the need for a standardized WMH assessment system and longitudinal follow-up cohort studies. A recent systematic review by Huang et al. (11) noted that approximately 60% of WMH volume variation remains unexplained by known cardiovascular risk factors, exposing limitations in the traditional paradigm of treating WMH as a single entity. Genetic studies further support this view: Patel et al. (15) identified WMH-associated genes enriched in oligodendrocytes and vascular endothelial cells via genome-wide association studies (GWAS), providing molecular evidence for the differential susceptibility of distinct brain regions to genetic and environmental factors, and revealing spatial specificity in microvascular structural and metabolic microenvironment damage. With advancements in neuroimaging techniques, spatial partitioning models based on tractography have enabled precise anatomical segmentation of white matter subregions (e.g., association fibers, projection fibers), propelling WMH research into a “subtype resolution” phase. For example, a recent study by Frauke et al. (16) demonstrated that frontoparietal WMH is associated with hypertension and genetic risk, whereas temporo-occipital WMH may reflect amyloid pathology. Collectively, these findings challenge the traditional global assessment paradigm, suggesting that it may obscure subtype-specific biological characteristics of WMH and necessitating deeper exploration of its spatial heterogeneity beyond conventional frameworks.

Against this backdrop, the present study aims to classify WMH imaging subtypes using intelligent automated segmentation techniques, analyze their independent risk factors via binary logistic regression, conduct volume quantitative analysis through multiple linear regression, systematically evaluate the spatial variability of WMH risk factors, and construct a risk stratification system. This approach will provide a novel paradigm for elucidating the heterogeneous pathological mechanisms of WMH, lay a theoretical foundation for developing precision prevention and treatment strategies targeting distinct anatomical subtypes, and offer imaging-based evidence for clinical precision intervention, thus holding significant theoretical and translational potential.

## Materials and methods

### Participants

**Figure 1:**
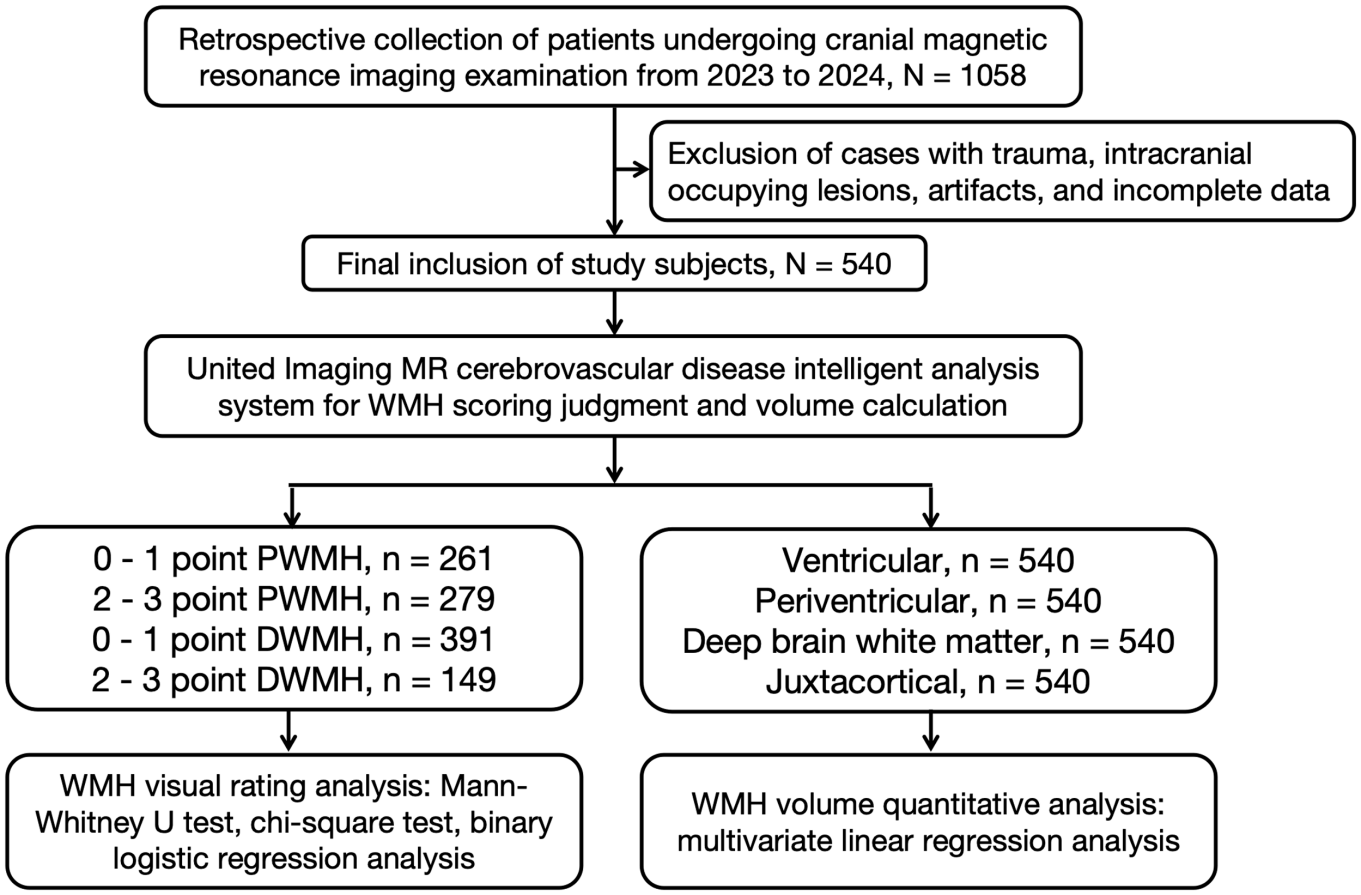
Study flowchart.

The flowchart for study inclusion is shown in Figure 1 all procedures and study protocols were approved by the Research Ethics Committee (Approval No. KY20240812-007). Due to the use of retrospective imaging data, the requirement for informed consent was waived by the Research Ethics Committee. This study was conducted in accordance with the principles of the Declaration of Helsinki. This study employed a cross-sectional design and retrospectively included inpatients or outpatients aged ≥40 years who were enrolled between January 1, 2023, and December 31, 2024, as study participants. Three sets of core data were collected: (1) baseline demographic data (age, sex); (2) medical history (hypertension, diabetes mellitus, hyperlipidemia, smoking history, and alcohol consumption history); and (3) laboratory indicators (e.g., serum uric acid, serum creatinine levels). Exclusion criteria included: ① imaging contraindications (history of acute craniocerebral trauma, intracranial hemorrhagic lesions); ② space-occupying lesions (neoplastic/non-neoplastic space-occupying lesions); and ③ technical interference factors (motion artifacts, susceptibility artifacts).

### MRI protocol

All imaging examinations were conducted using a United Imaging 3.0 T MRI scanner (uMR780) equipped with a 24-channel head–neck combined coil. Participants were positioned supine, with foam-rubber padding applied to minimize head motion. The standardized imaging protocol comprised: (1) T2-weighted imaging (T2WI): matrix size = 416 × 416, field of view (FOV) = 230 × 200 mm, repetition time/echo time (TR/TE) = 6044/128 ms, 23 slices with 5 mm thickness and 1 mm interslice gap; (2) T1-weighted fluid-attenuated inversion recovery (T1WI-FLAIR): matrix = 304 × 228, FOV = 230 × 200 mm, TR/TE = 2000/6.3 ms, matching slice configuration (23 slices, 5 mm thickness, 1 mm gap); (3) T2WI-FLAIR: matrix = 288 × 230, FOV = 230 × 200 mm, TR/TE = 8000/132 ms, identical slice parameters. Geometric accuracy was validated daily using a grid phantom (<1 mm distortion), with signal-to-noise ratio (SNR) maintained above 35 per NEMA MS-1 standards.

### Imaging processing

In this study, automated volumetric quantification of WMH and Fazekas scoring were performed on T2WI-FLAIR sequences using the United Imaging AI-based Cerebral Small Vessel Disease Analysis Module (UAI. OCR, Version R001) (17, 18). The automated segmentation results were independently reviewed by two senior neuroradiologists (X. F. Z. and Z. J., with 17 and 30 years of neuroimaging diagnostic experience, respectively) to ensure accuracy.

### Group stratification

**Figure 2:**
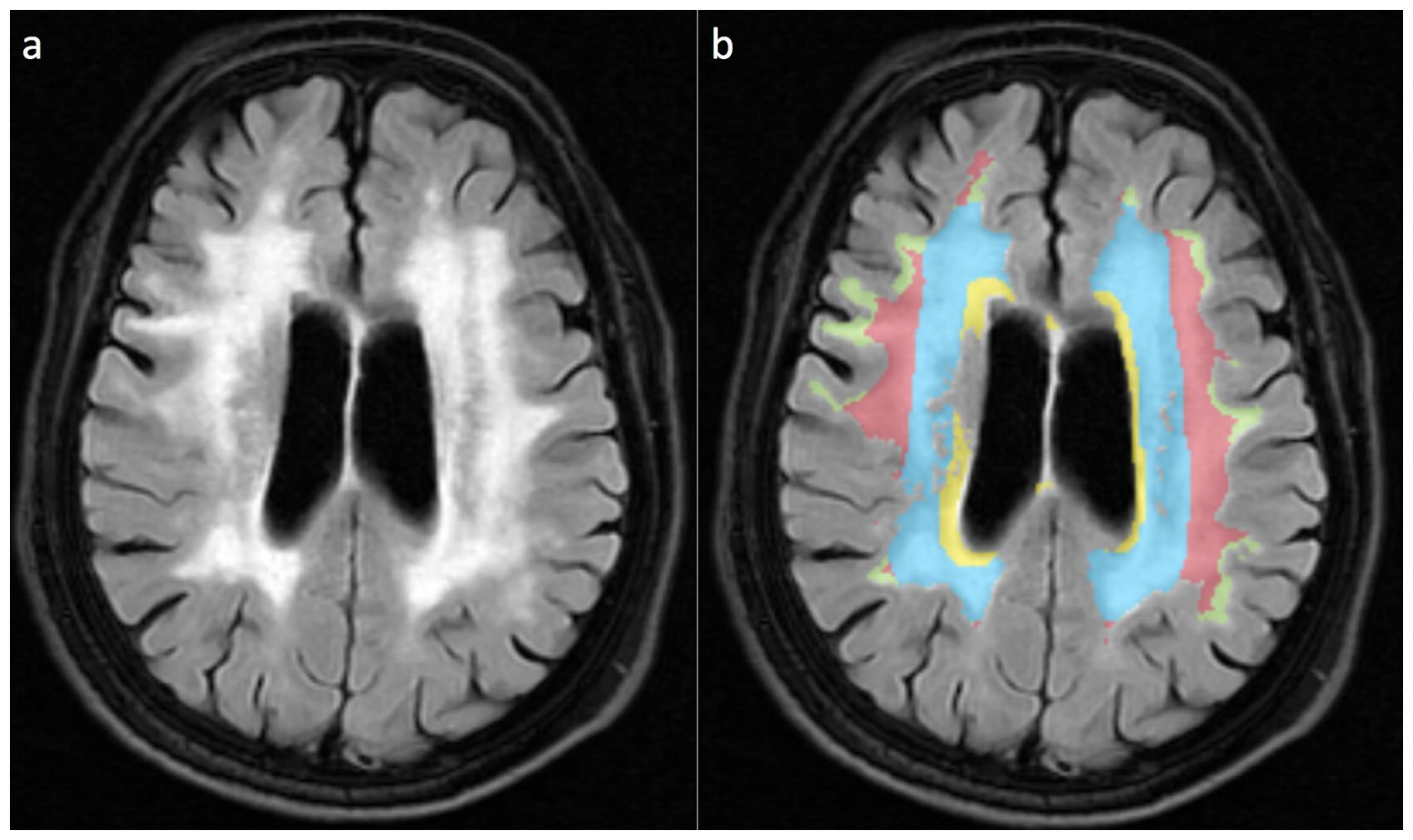
WMH is divided into four anatomical subregions based on the white matter topological localization system. **(a)** Schematic diagram of FLAIR sequences for different WMH subtypes; **(b)** Pseudo-color map corresponding to **(a)**, with the Ventricular region marked in yellow, the Periventricular zone displayed in blue, the Deep brain white matter region shown in red, and the Juxtacortical region in yellow-green.

This study implemented a dual-modality imaging assessment framework: for semi-quantitative visual analysis, PWMH and DWMH were graded using the Fazekas scoring system (19). PWMH severity was stratified as mild (grade 0–1: focal punctate or early confluent lesions) versus moderate–severe (grade 2–3: confluent lesions involving >25% of the lateral ventricular body circumference), while DWMH severity was classified as mild (grade 0–1: scattered punctate lesions <5 mm in diameter) versus moderate–severe (grade 2–3: extensive confluent lesions or bridging phenomena). For volumetric topographical analysis, WMHs were segmented into four anatomically defined subregions (Figure 2): (1) Ventricular (≤3 mm from ventricular walls) representing lesions abutting the ependymal surface; (2) Periventricular (3–13 mm) depicting lesions paralleling ventricular orientation; (3) DWMH spanning transitional areas between periventricular and juxtacortical regions; (4) Juxtacortical (≤4 mm from cortex,) marking lesions along corticomedullary junctions. Among them, the Ventricular rim and Periventricular zone are collectively referred to as PWMH.

### Statistical analyses

Statistical analyses and data visualization were performed using GraphPad Prism 9. Normality of quantitative variables (e.g., age, clinical biomarkers, volumetric measurements) was assessed via Shapiro–Wilk testing. For normally distributed data, distribution characteristics were described by Mean ± Standard Deviation (Mean ± SD), and intergroup comparisons were performed using the t-test; non-normally distributed variables were expressed as median and interquartile range [M (IQR)] and compared using Mann–Whitney U tests. Categorical variables (e.g., gender, disease subtypes, treatment groups) were presented as frequencies (percentages), with between-group differences analyzed by Chi-square tests.

To identify independent risk factors for moderate-to-severe WMH subtypes (dependent variable), binary logistic regression models were constructed with forward selection via the likelihood ratio (LR) method, incorporating candidate independent variables such as demographic characteristics, vascular risk factors, and imaging markers. For anatomical WMH subtype volumetric analyses, multiple linear regression with a stepwise approach (entry criteria *α* = 0.05; removal criteria *α* = 0.10) was implemented, where WMH subtype volumes served as dependent variables, and potential predictors (e.g., age, hypertension severity, white matter integrity metrics) were included as independent variables.

All statistical tests employed two-tailed thresholds (*p* < 0.05 for significance), with regression model outputs reported as standardized *β* coefficients and 95% confidence intervals (CIs).

## Results

### Demographic and clinical characteristics

The study cohort comprised 540 participants, with demographic characteristics and clinical parameter distributions detailed in Table 1. The age distribution was skewed, with a median age of 70.0 years (IQR: 59.0–77.0), and a balanced sex ratio (284 males, 52.6%). Regarding vascular risk factors, the prevalence of hypertension was 61.3% (331/540), with stage 3 hypertension accounting for the highest proportion (36.3%, 196 cases). Diabetes mellitus was present in 38.1% of participants (206 cases), among whom 14.6% (79 cases) had a disease duration exceeding 10 years. Metabolic profiling revealed hyperlipidemia in 49.8% of cases (269/540), with beta-2 microglobulin (β2-MG) abnormalities being the most common laboratory derangement (21.9%, 118 cases). Additional details are provided in Table 1.

**Table 1:** Demographic characteristics and clinical parameter distribution of the study cohort (*n* = 540).

Feature variable	Statistical description	Feature variable	Statistical description
Demographic characteristics		Metabolic diseases	
Age [M, (IQR)]	70.0 (59.0–77.0)	Type 2 diabetes (total cases)	206 (38.1%)
Sex distribution		Diabetes duration	
Male [*n* (%)]	284 (52.6%)	<5 years [n (%)]	46 (8.5%)
Female [*n* (%)]	256 (47.4%)	5–10 years [n (%)]	29 (5.4%)
Vascular risk factors		>10 years [n (%)]	79 (14.6%)
Hypertension (total cases)	331 (61.3%)	Unrecorded [n (%)]	52 (9.6%)
Grade 1 [*n* (%)]	38 (7.0%)	Laboratory Abnormalities	
Grade 2 [*n* (%)]	91 (16.9%)	Elevated uric acid [n (%)]	126 (23.3%)
Grade 3 [*n* (%)]	196 (36.3%)	Cys-C abnormality [n (%)]	109 (20.2%)
Other clinical parameters		Elevated creatinine [n (%)]	105 (19.4%)
Hyperlipidemia [*n* (%)]	269 (49.8%)	β2-MG abnormality [n (%)]	118 (21.9%)
Smoking history [*n* (%)]	178 (33.0%)	Cardiovascular Diseases	
Alcohol consumption [*n* (%)]	155 (28.7%)	Coronary heart disease [n (%)]	96 (17.8%)

M, Median; IQR, Interquartile range; Cys-C=Cystatin C; β2-MG = β2-microglobulin.

### Group comparison of risk factors for WMH stratified by Fazekas score

As shown in Table 2, Group comparisons revealed significant heterogeneity in risk factor distributions between PWMH and DWMH based on Fazekas score stratification. Common risk factors for higher Fazekas scores (2–3 vs. 0–1) in both subtypes included advanced age (PWMH: 73.0 vs. 62.0 years; DWMH: 72.0 vs. 68.0 years; both *p* < 0.0001), hypertension (PWMH: 75.6% vs. 46.0%; DWMH: 76.5% vs. 55.5%; both *p* < 0.0001), and renal dysfunction biomarkers (abnormal creatinine, Cys-C, and β2-MG; all *p* ≤ 0.003), suggesting shared mechanisms of chronic vascular injury and systemic metabolic dysregulation. Subtype-specific associations were also observed: PWMH severity showed stronger links to coronary heart disease (22.9% vs. 12.3%, *p* = 0.001), potentially reflecting heightened susceptibility of Periventricular regions to atherosclerotic ischemia, whereas DWMH severity was independently associated with smoking (40.3% vs. 30.2%, *p* = 0.03) and alcohol use (35.6% vs. 26.1%, *p* = 0.03), indicating neurotoxic vulnerability of deep white matter microvasculature. Additionally, DWMH exhibited stronger correlations with diabetes (47.0% vs. 34.8%, *p* = 0.009) and hyperuricemia (29.5% vs. 21.0%, *p* = 0.04) compared to PWMH (diabetes *p* = 0.04), likely mediated by synergistic blood–brain barrier disruption from insulin resistance and oxidative stress. Notably, a paradoxical subtype-specific association was observed for hyperlipidemia: no significant difference in prevalence was found between Fazekas 2–3 and 0–1 subgroups for PWMH (53.6% vs. 46.2%, *p* = 0.09), while DWMH severe subgroups paradoxically demonstrated lower hyperlipidemia rates (42.95% vs. 52.43%, *p* = 0.04).

**Table 2:** Group comparison of risk factors for WMH stratified by subtype and Fazekas score.

Variable	PWMH			DWMH		
	Score 0–1 (*n* = 261)	Score 2–3 (*n* = 279)	*p*	Score 0–1 (*n* = 391)	Score 2–3 (*n* = 149)	*p*
Age (years)	62.00(54.50 ~ 71.00)	73.00(67.00 ~ 80.00)	**<0.0001**	68.00(57.00 ~ 76.00)	72(65.50 ~ 79.00)	**<0.0001**
Sex distribution			0.28			0.61
Male [*n* (%)]	131 (50.19%)	153 (54.84%)		203(51.92%)	81(54.36%)	
Female [*n* (%)]	130 (49.81%)	126 (45.16%)		188(48.08%)	68(45.64%)	
Hypertension			**<0.0001**			**<0.0001**
Yes [*n* (%)]	120 (45.98%)	211 (75.63%)		217(55.50%)	114(76.51%)	
No [*n* (%)]	141 (54.02%)	68 (24.37%)		174(44.50%)	35(23.49%)	
Coronary heart disease			**0.001**			0.10
Yes [*n* (%)]	32 (12.26%)	64 (22.94%)		63(16.11%)	33(22.15%)	
No [*n* (%)]	229 (87.74%)	215 (77.06%)		328(83.89%)	116(77.85%)	
Diabetes mellitus			**0.04**			**0.009**
Yes [*n* (%)]	88 (33.72%)	118 (42.29%)		136(34.78%)	70(46.98%)	
No [*n* (%)]	173 (66.28%)	161 (57.71%)		255(65.32%)	79(53.02%)	
Smoking history			0.27			**0.03**
Yes [*n* (%)]	80 (30.65%)	98 (35.13%)		118(30.18%)	60(40.27%)	
No [*n* (%)]	181 (69.35%)	181 (64.87%)		273(69.82%)	89(59.73%)	
Alcohol consumption			0.19			**0.03**
Yes [*n* (%)]	68 (26.05%)	87 (31.18%)		102(26.09%)	53(35.57%)	
No [*n* (%)]	193 (73.95%)	192 (68.82%)		289(73.91%)	96(64.43%)	
Creatinine abnormality			**0.0001**			**0.003**
Yes [*n* (%)]	33 (12.64%)	72 (25.81%)		64(16.37%)	41(27.52%)	
No [*n* (%)]	228 (87.36%)	207 (74.19%)		327(83.63%)	108(72.48%)	
Uric acid abnormality			0.11			**0.04**
Yes [*n* (%)]	53 (20.31%)	73 (26.16%)		82(20.97%)	44(29.53%)	
No [*n* (%)]	208 (79.69%)	206 (73.84%)		309(79.03%)	105(70.47%)	
Cystatin C (Cys-C) abnormality			**0.003**			**0.0008**
Yes [*n* (%)]	39 (14.94%)	70 (25.09%)		65(16.62%)	44(29.53%)	
No [*n* (%)]	222 (85.06%)	209 (74.91%)		326(83.38%)	105(70.47%)	
β2-microglobulin (β2-MG) abnormality			**0.02**			**0.0008**
Yes [*n* (%)]	46 (17.62%)	72 (25.81%)		71(18.16%)	47(31.54%)	
No [*n* (%)]	215 (82.38%)	207 (74.19%)		320(81.84%)	102(68.46%)	
Hyperlipidemia			0.09			**0.04**
Yes [*n* (%)]	140 (53.64%)	129 (46.24%)		205(52.43%)	64(42.95%)	
No [*n* (%)]	121 (46.36%)	150 (53.76%)		186(47.57%)	85(57.05%)	

Bold values indicate statistically significant results (*p* < 0.05).

### Binary logistic regression analysis for identifying independent risk factors of moderate-to-severe WMH stratified by Fazekas score

**Figure 3:**
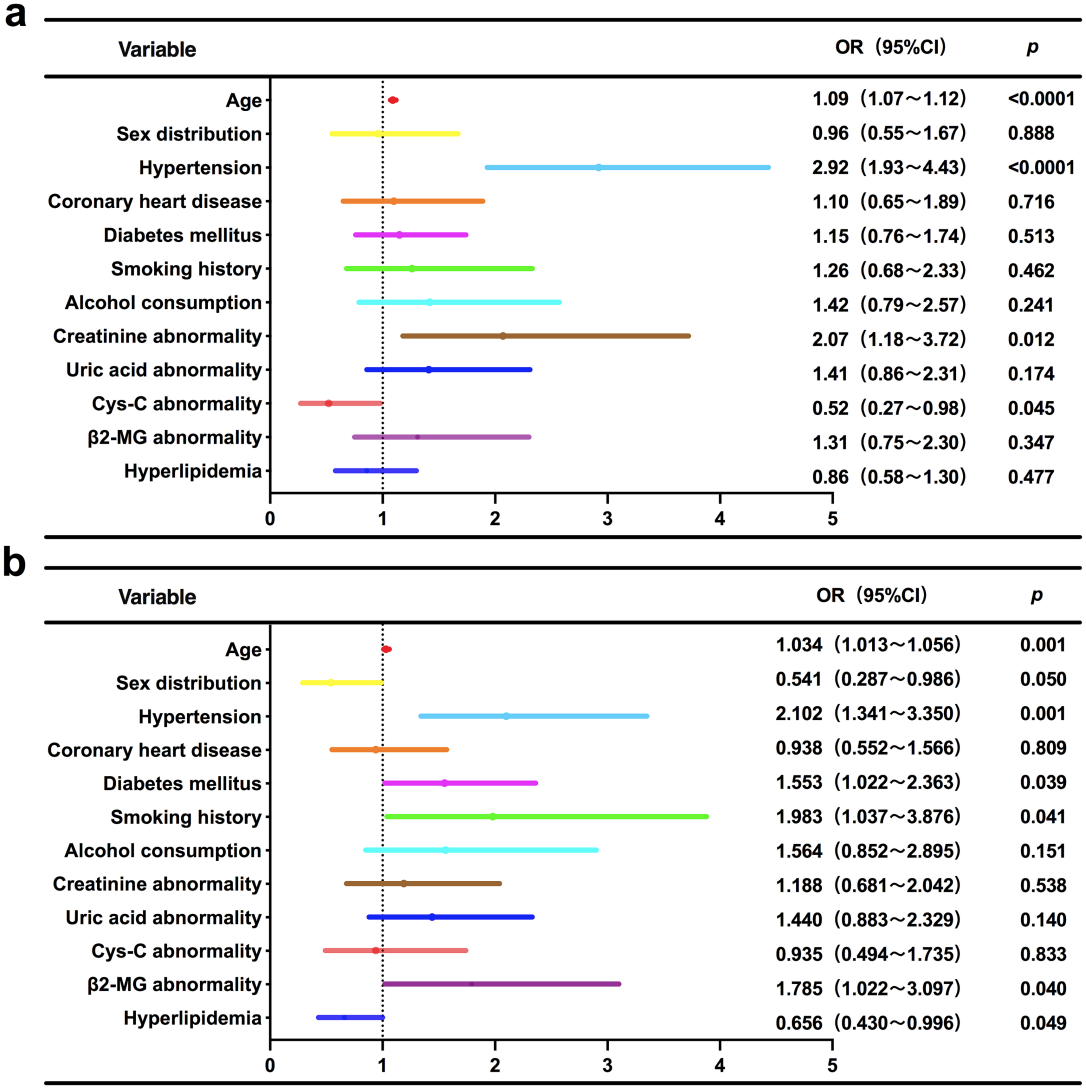
Forest plot of multivariable logistic regression analysis for independent risk factors associated with moderate-to-severe Fazekas score WMH subtypes. **(a)** PWMH; **(b)** DWMH.

As shown in Table 3 and Figure 3, Multivariable regression revealed significant heterogeneity in risk profiles between PWMH and DWMH with moderate-to-severe Fazekas scores (2, 3). Common drivers included aging (PWMH: OR = 1.09 per year, 95%CI 1.07–1.12, *p* < 0.0001; DWMH: OR = 1.034 per year, 95%CI 1.013–1.056, *p* = 0.001) and hypertension (PWMH: OR = 2.92, *p* < 0.0001; DWMH: OR = 2.10, *p* = 0.001), underscoring their central roles in WMH progression. Subtype-specific patterns emerged: PWMH severity uniquely correlated with creatinine abnormalities (OR = 2.07, *p* = 0.012) and paradoxically with lower cystatin C (Cys-C) levels (OR = 0.52, *p* = 0.045). This inverse Cys-C-PWMH association may reflect impaired periventricular blood–brain barrier integrity, dysregulated cerebrospinal fluid clearance, or confounding by cystatin-mediated cysteine protease inhibition, statin use, or population-specific comorbidities. Conversely, DWMH risk was independently driven by smoking (OR = 1.98, *p* = 0.041), diabetes (OR = 1.55, *p* = 0.039), and β2-microglobulin abnormalities (OR = 1.79, *p* = 0.040), highlighting deep white matter vulnerability to oxidative stress and glucotoxicity. Sex-specific protection (male: OR = 0.54, *p* = 0.05) and inverse dyslipidemia association (OR = 0.66, *p* = 0.049) further highlighted metabolic-microvascular regulatory heterogeneity.

**Table 3:** Independent risk factor analysis for moderate-to-severe Fazekas-scored WMH across subtypes.

Variable	PWMH			DWMH		
	*β*	OR(95%CI)	*p*	*β*	OR(95%CI)	*p*
Age (per year)	0.088	1.09 (1.07–1.12)	**<0.0001**	0.033	1.034(1.013 ~ 1.056)	**0.001**
Sex (Male)	−0.039	0.96 (0.55–1.67)	0.888	−0.615	0.541(0.287 ~ 0.986)	0.050
Hypertension	1.070	2.92 (1.93–4.43)	**<0.0001**	0.743	2.102(1.341 ~ 3.350)	**0.001**
Coronary heart disease	0.099	1.10 (0.65–1.89)	0.716	−0.064	0.938(0.552 ~ 1.566)	0.809
Diabetes mellitus	0.138	1.15 (0.76–1.74)	0.513	0.440	1.553(1.022 ~ 2.363)	**0.039**
Smoking history	0.230	1.26 (0.68–2.33)	0.462	0.685	1.983(1.037 ~ 3.876)	**0.041**
Alcohol consumption	0.352	1.42 (0.79–2.57)	0.241	0.447	1.564(0.852 ~ 2.895)	0.151
Creatinine abnormality	0.732	2.07 (1.18–3.72)	**0.012**	0.172	1.188(0.681 ~ 2.042)	0.538
Uric acid abnormality	0.341	1.41 (0.86–2.31)	0.174	0.365	1.440(0.883 ~ 2.329)	0.140
Cys-C abnormality	−0.657	0.52 (0.27–0.98)	**0.045**	−0.067	0.935(0.494 ~ 1.735)	0.833
β2-MG abnormality	0.088	1.09 (1.07–1.12)	0.347	0.579	1.785(1.022 ~ 3.097)	**0.040**
Hyperlipidemia	−0.039	0.96 (0.55–1.67)	0.477	−0.422	0.656(0.430 ~ 0.996)	**0.049**

Bold values indicate statistically significant results (*p* < 0.05).

### Multivariable linear regression analysis of risk factors for WMH volumes based on anatomical subtypes

This study systematically evaluated the effects of demographic characteristics, vascular risk factors, and laboratory parameters on the volumetric distribution of WMH subtypes using multivariate linear regression models (Table 4). Models incorporating age, sex, hypertension, coronary artery disease, diabetes, smoking history, alcohol consumption, creatinine abnormalities, uric acid dysregulation, Cys-C anomalies, *β*2-MG abnormalities, and dyslipidemia were constructed to assess Ventricular, Periventricular, Juxtacortical, and DWMH volumes. The analysis revealed significant spatial heterogeneity in risk factor profiles across WMH subtypes: advancing age exhibited positive associations with ventricular (*β* = 0.085, 95% CI 0.062–0.108, *p* < 0.0001), Periventricular (*β* = 0.236, 95% CI 0.201–0.271, *p* < 0.0001), and Juxtacortical volumes (*β* = 0.010, 95% CI 0.003–0.017, *p* = 0.005), identifying aging as a central driver of WMH progression. Hypertension demonstrated spatially graded effects, with diminishing impact from Ventricular to cortical regions, significantly elevating Ventricular (*β* = 1.209, 95% CI 0.893–1.525, *p* < 0.0001), Periventricular (*β* = 3.618, 95% CI 2.874–4.362, *p* < 0.0001), and Juxtacortical volumes (*β* = 0.170, 95% CI 0.013–0.327, *p* = 0.034). Cys-C abnormalities inversely correlated with Periventricular WMH (*β* = −0.917, 95% CI -3.512–1.678, *p* = 0.473), aligning with logistic regression findings of reduced PWMH severity risk. Notably, diabetes selectively exacerbated Periventricular WMH expansion (*β* = 3.073, 95% CI 0.568–5.578, *p* = 0.016), potentially reflecting microvascular susceptibility to glycemic dysregulation in this region. Coronary artery disease (*β* = 0.232, 95% CI 0.034–0.430, *p* = 0.022) and creatinine abnormalities (*β* = 0.280, 95% CI 0.070–0.490, *p* = 0.009) preferentially promoted Juxtacortical pathology, suggesting that cardiorenal comorbidities may exacerbate subcortical damage through blood–brain barrier leakage. Intriguingly, dyslipidemia showed inverse associations with Juxtacortical (*β* = −0.194, 95% CI -0.347–-0.041, *p* = 0.011) and DWMH volumes (*β* = −0.783, 95% CI -1.296–-0.270, *p* = 0.003), further substantiating the necessity of integrating anatomical-metabolic interaction networks to decipher WMH spatial heterogeneity.

**Table 4:** Multivariable linear regression analysis of risk factors for WMH volumes based on anatomical subtypes.

Variable	Ventricular			Periventricular			Juxtacortical			DWMH		
	*β*	*t*	*p*	*β*	*t*	*p*	*β*	*t*	*p*	*β*	*t*	*p*
Age (per year)	0.085	7.021	****	0.236	6.134	****	0.010	2.850	**0.005**	0.018	1.457	0.146
Sex (Male)	0.200	0.569	0.570	−0.469	0.420	0.675	−0.148	1.418	0.157	−0.458	1.276	0.202
Hypertension	1.209	4.501	****	3.618	4.239	****	0.170	2.127	**0.034**	0.537	1.959	0.051
Coronary heart disease	−0.002	0.006	0.995	0.636	0.589	0.556	0.232	2.296	**0.022**	0.076	0.218	0.827
Diabetes mellitus	0.695	1.743	0.082	3.073	2.426	**0.016**	0.152	1.283	0.200	0.738	1.813	0.070
Smoking history	0.479	1.252	0.211	0.708	0.582	0.561	0.11	1.027	0.305	0.414	1.060	0.290
Alcohol consumption	0.132	0.496	0.620	0.240	0.284	0.776	0.051	0.641	0.522	0.439	1.619	0.106
Creatinine abnormality	0.517	1.439	0.151	1.714	1.503	0.133	0.280	2.626	**0.009**	0.713	1.945	0.052
Uric acid abnormality	0.564	1.825	0.069	1.385	1.412	0.159	0.086	0.940	0.348	0.363	1.150	0.251
Cys-C abnormality	0.158	0.392	0.695	−0.917	0.718	0.473	−0.117	0.980	0.327	−0.005	0.013	0.990
β2-MG abnormality	0.378	0.287	0.287	−0.433	0.384	0.701	0.149	1.412	0.159	0.378	1.045	0.297
Hyperlipidemia	−0.274	0.286	0.286	−1.088	1.335	0.183	−0.194	2.540	**0.011**	−0.783	2.988	**0.003**

****p < 0.0001. Bold values indicate statistically significant results (*p* < 0.05).

## Discussion

WMH, as a core imaging biomarker of cerebral small vessel disease (20), has been widely recognized for its value in the early warning of cerebrovascular and neurodegenerative diseases. However, existing studies often treat WMH as a single pathological entity, and the traditional paradigm based on overall volume or semi-quantitative scoring (e.g., Fazekas scale) struggles to reveal its spatial heterogeneity and differential pathological mechanisms. This limitation leads to controversies in risk factor assessment, unclear therapeutic targets, and remains a critical research bottleneck in neuroimaging that urgently requires breakthroughs. This study, through a FLAIR-MRI imaging subtype classification system combined with multimodal logistic regression and multiple linear regression analyses, systematically characterized the heterogeneity of risk factors between PWMH and DWMH. Although both subtypes share fundamental driving factors such as age and hypertension, PWMH was more significantly associated with coronary heart disease and abnormal renal function markers (e.g., creatinine), whereas DWMH was independently driven by metabolic-microvascular interactive factors including smoking, diabetes mellitus, and β2-microglobulin abnormalities. Notably, hyperlipidemia showed a negative association with DWMH severity. These findings transcend the limitations of traditional global assessment and provide critical evidence for elucidating the pathological mechanisms underlying WMH spatial heterogeneity, constructing an imaging subtype-based risk stratification system, and developing targeted intervention strategies.

The core scientific significance of this study lies in revealing the differential risk factor-driven patterns of WMH across distinct imaging subtypes. The results indicate that the spatial heterogeneity of WMH may stem from the specific injury mechanisms involving microvascular structures and metabolic microenvironments in different brain regions. First, age and hypertension, as fundamental driving factors, exert broad-spectrum effects on the overall WMH burden, while diabetes, coronary heart disease, and other conditions amplify lesion heterogeneity by selectively targeting specific anatomical regions. Second, the strong association between the PWMH subtype and renal function markers suggests that this subtype may reflect the pathological process of systemic damage along the brain-kidney axis, where glomerular filtration dysfunction may exacerbate periventricular white matter microcirculatory disturbances through pathways such as uremic toxin accumulation and endothelial damage. The DWMH subtype, in contrast, more likely represents the synergistic effects of local metabolic homeostasis imbalance and capillary dysfunction within the brain parenchyma, where oxidative stress induced by smoking and diabetes may promote deep white matter ischemic injury by disrupting the integrity of the neurovascular unit. Additionally, the selective promotion of juxtacortical WMH volume by coronary heart disease and creatinine abnormalities further confirms the correspondence between WMH spatial distribution and underlying etiologies. Finally, the observed negative association between hyperlipidemia and WMH severity requires cautious interpretation, suggesting that future research should transcend traditional biochemical indicator classification frameworks and establish a precision evaluation system based on lipoprotein subtypes and WMH. In summary, these findings collectively validate the scientific value of the “spatial classification-mechanism analysis-precision intervention” research paradigm for WMH, providing theoretical evidence for its pathological classification and individualized prevention strategies—PWMH management should focus on optimizing cerebrospinal fluid dynamics and monitoring brain-kidney axis comorbidities, while DWMH emphasizes targeting metabolic-microvascular networks. Additionally, integrating WMH spatial classification into radiological diagnostic criteria is recommended to guide precise clinical decision-making.

The data from this study confirm that the heterogeneity of risk factors for WMH not only shares core mechanisms with previous research (e.g., the vascular damage effect of hypertension) but also presents inconsistencies due to population characteristics (e.g., high metabolic burden in hospitalized patients), indicator definitions (e.g., composite hyperlipidemia), and analytical methods (differences between multivariate logistic regression and multiple linear regression). Specifically, aging significantly increased the risk of severe WMH in both PWMH (OR = 1.09 per year, 95%CI 1.07–1.12, *p* < 0.0001) and DWMH (OR = 1.034 per year, 95%CI 1.013–1.056, *p* = 0.001). Hypertension exerted spatially gradient risk amplification effects on PWMH (OR = 2.92) and DWMH (OR = 2.10) (both *p* < 0.001). Multiple linear regression further revealed that aging (*β* = 0.236) and hypertension (*β* = 3.618) had the strongest driving effects on Periventricular WMH volume, with the impact intensity of hypertension on Periventricular regions being 21 times greater than that on Juxtacortical regions (*β* = 3.618 vs. 0.170, both *p* < 0.05). These findings corroborate the cross-regional consensus on aging and hypertension as core risk factors for WMH from both visual scoring and volume quantification perspectives (5, 6, 21), while also revealing the spatial heterogeneity of WMH—hypertension exerts stronger targeted damage on Periventricular white matter. Notably, the significant promotive effect of aging and hypertension on Periventricular WMH volume (with β values as high as 3.618) in this study conflicts with Fatemeh et al.’s research (21), which suggested that traditional cardiovascular risk factors explained only 15% of WMH volume variation, with aging and undefined mechanisms dominating. This discrepancy may be attributed to their use of whole-brain WMH volume as a holistic indicator, whereas our study revealed spatially specific drivers through imaging subtype analysis, suggesting that imaging subtype classification can more precisely quantify the contribution of risk factors. Additionally, creatinine abnormalities significantly increased the proportion of moderate-to-severe PWMH (Fazekas scores 2–3) (25.81% vs. 12.64%, *p* < 0.0001) and moderate-to-severe DWMH (27.52% vs. 16.37%, *p* = 0.003). Multivariate logistic regression confirmed creatinine abnormalities as an independent risk factor for moderate-to-severe PWMH (OR = 2.07, 95%CI:1.18–3.72, *p* = 0.012), but no significant effect was observed for DWMH (*p* = 0.538). Quantitative MRI studies (22) indicated that Periventricular white matter of the brain exhibited higher free water content and plasma volume, supporting the pathological features of more pronounced microvascular leakage and interstitial edema in this region. This study partially aligns with Zhang et al.’s conclusion (7) that proteinuria is an independent risk factor for WMH severity in non-stroke elderly populations, but further multiple linear regression revealed that creatinine abnormalities selectively promoted Juxtacortical WMH volume (*β* = 0.280, *t* = 2.626, *p* = 0.009), while their effects on Ventricular (*β* = 0.517, *p* = 0.151) and Periventricular (*β* = 1.714, *p* = 0.133) regions were not statistically significant. This suggests that “brain-kidney axis” toxin accumulation may differentially impair blood–brain barrier permeability across brain regions, providing pathological evidence for WMH spatial classification based on systemic brain-kidney axis damage and refining the specific association between renal function markers and WMH anatomical subtypes. Another notable finding is the inconsistency between this study’s negative association of hyperlipidemia with WMH severity and previous research (11, 23). This study showed that the hyperlipidemia prevalence in moderate-to-severe DWMH groups was significantly lower than in mild DWMH groups (42.95% vs. 52.43%, *p* = 0.04). Multivariate logistic regression further indicated a negative correlation between hyperlipidemia and moderate-to-severe DWMH lesion risk (OR = 0.656, 95%CI:0.430–0.996, *p* = 0.049), while multiple linear regression demonstrated significant negative effects of hyperlipidemia on DWMH (*β* = −0.783, *p* = 0.003) and Juxtacortical WMH volumes (*β* = −0.194, *p* = 0.011). This phenomenon may arise from the composite hyperlipidemia definition used in this study (defined by meeting any criterion of elevated triglycerides, total cholesterol, LDL-C, or reduced HDL-C), which masks the interplay between the protective effects of HDL-C and pro-inflammatory effects of LDL-C. Additionally, the exclusion of confounding factors such as statin use and the baseline high metabolic disorder risk in hospitalized patients (e.g., 38.1% diabetes prevalence, 61.3% hypertension) weakened the association between traditional lipid indicators and WMH. These findings suggest that future studies should integrate lipoprotein subtypes (e.g., oxidized LDL, HDL-C functional subclasses) and statin exposure history to dissect the differential impacts of lipid components on distinct WMH anatomical subtypes, transcending the singular notion that “hyperlipidemia = increased risk” and providing a basis for precise lipid management. The selective driving effects of diabetes and coronary heart disease on WMH subtypes also warrant emphasis: diabetes was found to specifically promote DWMH volume (*β* = 0.89, *p* = 0.02), while coronary heart disease selectively increased Juxtacortical WMH volume (*β* = 0.232, *p* = 0.022). Notably, the association between diabetes and DWMH volume aligns closely with previous research in both pathological mechanisms and clinical phenotypes. Patel et al.’s GWAS-based study (15) revealed that WMH-related genetic signals are enriched in oligodendrocytes, suggesting that abnormal glucose metabolism may exacerbate deep white matter ischemia through mitochondrial dysfunction in glial cells, further disrupting blood–brain barrier homeostasis and aggravating white matter damage. Clinical studies (24) have also confirmed that in patients with diabetes, DWMH volume is significantly higher than in non-diabetic patients (*β* = 0.89 vs. 0.57, *p* = 0.02) and positively correlated with insulin resistance indices (*r* = 0.35, *p* < 0.001). Imaging analyses further revealed (25) that deep white matter lesions in diabetic patients are predominantly distributed in the frontoparietal-striatal circuit, with lesion morphological irregularity (concavity index *β* = 0.06, *p* = 0.01) aligning with the metabolic-microvascular interactive damage mechanism. Collectively, these evidence support the hypothesis that diabetes may synergistically drive DWMH progression through oxidative stress and capillary leakage.

This study elucidates the spatial heterogeneity of WMH and its correspondence with multisystem pathological networks, transcending previous limitations that conceptualized WMH as a single pathological entity. The novel spatial typing model of WMH establishes a paradigm shift for personalized prevention and management of cerebral small vessel disease, with clinical implications manifested in three domains: (1) Subtype-guided precision intervention: For PWMH, prioritized interventions should target cerebrospinal fluid dynamics disturbances and circadian blood pressure regulation, while incorporating creatinine monitoring to evaluate brain-kidney axis comorbidity risks, thereby complementing the traditional “single-vessel disease management” model. For DWMH, clinical focus should emphasize the metabolic-microvascular interaction network, advocating concurrent assessment of carotid intima-media thickness (IMT) and homeostasis model assessment of insulin resistance (HOMA-IR) in risk stratification. (2) Refinement of neuroimaging criteria: Current semi-quantitative WMH scoring systems (e.g., Fazekas scale) based on FLAIR-MRI fail to discriminate pathological heterogeneity between periventricular and deep lesions. Our findings demonstrate that spatial phenotype characterization - including confluent lesions in PWMH and punctate distributions in DWMH - facilitates differentiation between arteriolosclerosis-dominant and blood–brain barrier leakage-dominant subtypes. We therefore recommend incorporating WMH subtype classification annotations in radiological reports to optimize clinical decision-making. (3) Re-evaluation of lipid management strategies: The paradoxical association between hyperlipidemia and WHM severity underscores limitations in current diagnostic criteria. Clinical practice should integrate lipid subfraction analysis (e.g., oxidized LDL quantification and HDL-C functional assessment) with APOE genotyping to cautiously balance the neuroprotective effects and cognitive risks of statin therapy in DWMH patients.

The limitations of this study encompass the following aspects: First, the exclusive enrollment of hospitalized patients may introduce selection bias, potentially limiting the generalizability of findings. Second, the absence of cerebrospinal fluid (CSF) biomarkers [e.g., glial fibrillary acidic protein (GFAP) and neurofilament light chain] precludes direct validation of the hypothesis that CSF hydrodynamic abnormalities mediate interstitial fluid metabolic disturbances. Third, critical covariates including educational attainment, occupational physical activity, disease duration of hypertension/diabetes, smoking intensity, and alcohol consumption levels – all potential modifiers of WMH pathogenesis and progression – were not incorporated into statistical analyses. Fourth, the observed “protective effect” of hyperlipidemia might be confounded by statin usage and other variables, necessitating future verification through longitudinal cohorts combined with multimodal imaging (e.g., dynamic contrast-enhanced MRI for blood–brain barrier permeability assessment) and single-cell transcriptomics to delineate spatiotemporal evolutionary patterns and molecular regulatory networks underlying WMH subtypes.

## Conclusion

This study revealed the spatial heterogeneity and differential pathological mechanisms of WMH through imaging subtype analysis: Although both PWMH and DWMH are driven by baseline factors such as age and hypertension, their risk profiles exhibit significant differences—PWMH is more prominently associated with coronary heart disease and renal dysfunction, while DWMH is independently driven by metabolic-microvascular interactive factors including smoking, diabetes mellitus, and abnormal β2-microglobulin. These findings support the use of spatial classification as a key tool for optimizing WMH risk stratification, provide guidance for subtype-specific clinical interventions in WMH, and lay a theoretical foundation for the individualized prevention and treatment of cerebral small vessel disease. Future research should validate the prognostic efficacy of the subtyping model through multicenter longitudinal cohorts, and integrate techniques such as dynamic contrast-enhanced MRI and single-cell transcriptomics to elucidate the evolutionary mechanisms of subtypes, thereby promoting the transformation of cerebral small vessel disease prevention and treatment toward precision medicine.

## Data Availability

The original contributions presented in the study are included in the article/supplementary material, further inquiries can be directed to the corresponding author/s.

## References

[1] Wei L Zhao X Luo J Xiao M Li B Zhu Z. White matter hyperintensity is associated with malignant cerebral edema in ischemic stroke treated with thrombectomy. J Magn Reson Imaging. (2025) 61:441–9. doi: 10.1002/jmri.29423, PMID: 38722187

[2] Wang X Lyu J Meng Z Wu X Chen W Wang G. Small vessel disease burden predicts functional outcomes in patients with acute ischemic stroke using machine learning. CNS Neurosci Ther. (2023) 29:1024–33. doi: 10.1111/cns.14071, PMID: 36650639 PMC10018092

[3] Zhao X Zuo M Zhan F Fan P Liu S Taylor M. Cognition mediates the relationship between white matter hyperintensity and motor function in patients with cerebral small vessel disease: a cross-sectional study. Quant Imaging Med Surg. (2024) 14:1058. doi: 10.21037/qims-24-1058, PMID: 39429558 PMC11485344

[4] Xiufu Z Ruipeng L Jun Z Yonglong L Yulin W Jian Z. Analysis of influencing factors of early neurological improvement after intravenous rt-PA thrombolysis in acute anterior circulation ischemic stroke. Front Neurol. (2022) 13:1037663. doi: 10.3389/fneur.2022.1037663, PMID: 36324389 PMC9619649

[5] You Z Ma S Xu H Wu Z You Z. Comorbidity of white matter lesions in Parkinson's disease: a study on risk factors and phenotypic differences. Neurol Sci. (2024) 46:7735. doi: 10.1007/s10072-024-07735-x, PMID: 39214869

[6] Li J Tian Y Shi Y Cui Y Lian J Liu P. Association of vulnerable plaques with white matter hyperintensities on high-resolution magnetic resonance imaging. Quant Imaging Med Surg. (2024) 14:1856. doi: 10.21037/qims-23-1856, PMID: 38720851 PMC11074730

[7] Zheng K Wang Z Chen X Chen J Fu Y Chen Q. Analysis of risk factors for white matter Hyperintensity in older adults without stroke. Brain Sci. (2023) 13:835. doi: 10.3390/brainsci13050835, PMID: 37239307 PMC10216050

[8] Lim C Lee H Moon Y Han S-H Kim H Chung H. Volume and permeability of white matter hyperintensity on cognition: a DCE imaging study of an older cohort with and without cognitive impairment. J Magn Reson Imaging. (2024) 61:2260–70. doi: 10.1002/jmri.29631, PMID: 39425583 PMC11987793

[9] Shu L Zhong K Chen N Gu W Shang W Liang J. Predicting the severity of white matter lesions among patients with cerebrovascular risk factors based on retinal images and clinical laboratory data: a deep learning study. Front Neurol. (2023) 14:1168836. doi: 10.3389/fneur.2023.1168836, PMID: 37492851 PMC10363667

[10] Nezu T Eto F Hironaka A Aoki S Neshige S Tasaka S. Vagus nerve size determined via ultrasonography is associated with white matter lesions in patients with vascular risk factors. J Ultrasound. (2024) 27:723–32. doi: 10.1007/s40477-024-00936-2, PMID: 39073732 PMC11333691

[11] Huang W-Q Lin Q Tzeng C-M. Leukoaraiosis: epidemiology, imaging, risk factors, and management of age-related cerebral white matter hyperintensities. J Stroke. (2024) 26:131–63. doi: 10.5853/jos.2023.02719, PMID: 38836265 PMC11164597

[12] Nemati S Hosseinpoor N Khanhakimi M Arzpeyma S Ghaffari M Mostafavi S. White matter lesions in brain MRI and cardiovascular risk factors in sudden sensorineural hearing loss patients: a comparative study. Am J Otolaryngol. (2025) 46:104607. doi: 10.1016/j.amjoto.2025.104607, PMID: 40088764

[13] al-Hashel J Alroughani R Gad K al-Sarraf L Ahmed S. Risk factors of white matter hyperintensities in migraine patients. BMC Neurol. (2022) 22:159. doi: 10.1186/s12883-022-02680-8, PMID: 35488255 PMC9052543

[14] Ko J Choi Y Jeong E Kim H-J Lee G Park J. Automated quantification of cerebral microbleeds in SWI: association with vascular risk factors, white matter hyperintensity burden, and cognitive function. AJNR Am J Neuroradiol. (2024) 46:1007–15. doi: 10.3174/ajnr.A8552, PMID: 39443150 PMC12091992

[15] Patel Y Shin J Sliz E Tang A Mishra A Xia R. Genetic risk factors underlying white matter hyperintensities and cortical atrophy. Nat Commun. (2024) 15:689. doi: 10.1038/s41467-024-53689-1, PMID: 39496600 PMC11535513

[16] Beyer F Tsuchida A Soumaré A Rajula HSR Mishra A Crivello F. White matter hyperintensity spatial patterns: risk factors and clinical correlates. Alzheimers Dement. (2025) 21:e70053. doi: 10.1002/alz.70053, PMID: 40189793 PMC11972985

[17] Zhang Z Ding Z Chen F Hua R Wu J Shen Z. Quantitative analysis of multimodal MRI markers and clinical risk factors for cerebral small vessel disease based on deep learning. Int J Gen Med. (2024) 17:739–50. doi: 10.2147/IJGM.S446531, PMID: 38463439 PMC10923240

[18] Zhu W Huang H Zhou Y Shi F Shen H Chen R. Automatic segmentation of white matter hyperintensities in routine clinical brain MRI by 2d VB-net: a large-scale study. Front Aging Neurosci. (2022) 14:15009. doi: 10.3389/fnagi.2022.915009, PMID: 35966772 PMC9372352

[19] Fazekas F Chawluk JB Alavi A Hurtig HI Zimmerman RA. MR signal abnormalities at 1.5 T in Alzheimer's dementia and normal aging. AJR Am J Roentgenol. (1987) 149:351–6. doi: 10.2214/ajr.149.2.351, PMID: 3496763

[20] Arboix A Massons J García-Eroles L Targa C Comes E Parra O. Nineteen-year trends in risk factors, clinical characteristics and prognosis in lacunar infarcts. Neuroepidemiology. (2010) 35:231–6. doi: 10.1159/000319460, PMID: 20861654

[21] Khosravi F Hosseini LE Mohammadi SH. Contribution of conventional cardiovascular risk factors to brain white matter hyperintensities. J Am Heart Assoc. (2023) 12:e030676. doi: 10.1161/JAHA.123.030676, PMID: 37421292 PMC10382123

[22] Voorter PHM Stringer MS van Dinther M Kerkhofs D Dewenter A Blair GW. Heterogeneity and penumbra of white matter hyperintensities in small vessel diseases determined by quantitative MRI. Stroke. (2024) 56:128–37. doi: 10.1161/STROKEAHA.124.047910, PMID: 39648904

[23] Antoine G C G. White matter hyperintensities in Alzheimer's disease: beyond (but not instead of) the vascular contribution. Alzheimers Dement. (2023) 19:4262–3. doi: 10.1002/alz.13372, PMID: 37437034

[24] Keller AJ Kant IMJ Slooter AJC van S van M van M. Different cardiovascular risk factors are related to distinct white matter hyperintensity MRI phenotypes in older adults. Neuroimage Clin. (2022) 35:103131. doi: 10.1016/j.nicl.2022.103131, PMID: 36002958 PMC9421504

[25] Lim J-S Lee K-J Kim BJ Ryu WS Chung J Gwak DS. Nonhypertensive white matter Hyperintensities in stroke: risk factors, neuroimaging characteristics, and prognosis. J Am Heart Assoc Cardiovasc Cerebrovasc Dis. (2023) 12:12. doi: 10.1161/JAHA.123.030515 PMC10727348 38014679

